# Telemedicine as a Strategic Tool to Enhance the Effectiveness of Care Processes: Technological and Regulatory Evolution over the Past Two Decades

**DOI:** 10.3390/healthcare11050734

**Published:** 2023-03-02

**Authors:** Giovanna Ricci, Anna Maria Caraffa, Filippo Gibelli

**Affiliations:** Section of Legal Medicine, School of Law, University of Camerino, 62032 Camerino, Italy

## 1. Introduction

Digital innovation represents one of the largest areas of investment in healthcare. The application of technologies pertaining to the ever-evolving infrastructure of information and communication technology (ICT) aims to making the many processes and services related to healthcare delivery more effective and efficient. In the complex and articulated panorama of e-health, telemedicine certainly represents one of the key strategic tools to support and improve numerous care processes. For many of these processes, distance is a critical factor for access to care. For this reason, e-health has become the subject of numerous interventions and initiatives by international policies for decades now.

In a communication, made on 4 November 2008, on “telemedicine for the benefit of patients, health systems, and society”, the European Commission highlighted the substantial contributions that telemedicine could make to the quality of life of its citizens, e.g., improving access to healthcare in areas that are difficult to access or that have a shortage of qualified personnel; reducing hospitalizations for people with chronic diseases through telemonitoring; and reducing waiting lists for some diagnostic services (e.g., teleradiology). Many of these methods, consequently, would also lead to benefits from an economic point of view (optimization of resources and increased productivity) [1,2]. Being aware of the high degree of fragmentation in the deployment of telemedicine services, the communication aimed to encourage member states to integrate telemedicine services into their health policies by identifying actions to be taken, such as building confidence in telemedicine services and fostering their acceptance by health professionals, patients, and health authorities; providing legal clarity; solving technical problems; and facilitating market development. In addition, the communication from the Commission pointed out that public acceptance of and trust in telemedicine systems are highly dependent on the acceptance and trust that health professionals themselves develop with regard to such tools. Therefore, it becomes essential to “enhance the dissemination of evidence regarding the effectiveness of telemedicine services, their safety features, and their ease of use” [1].

It also becomes necessary to address ethical issues, particularly the repercussions to the doctor–patient relationship, the principles of confidentiality, and the protection of personal data, in compliance with existing regulations [3]. The European Economic and Social Committee welcomes with interest the actions proposed by the Commission: “without prejudice to respect for the principle of subsidiarity. Member States remain responsible for their public health policy as well as the development of telemedicine, according to their investment capacities” [4]. In its conclusions, the Committee defines telemedicine as “a kind of cultural revolution, which therefore requires appropriate communication. New professions may develop to accompany this evolution. The Committee believes that the development of telemedicine should be seen in the context of an overall evolution of health policies and systems. Health system users will have to become more “actors” in their own health”.

For decades now, telemedicine has been the subject of numerous normative acts, projects, and strategic documents at the European level, with many countries embracing and widely implementing it across various areas of application. In Italy, there are many telemedicine initiatives, although they are equally fragmented and uneven. The advent of the COVID-19 pandemic significantly accelerated the national spread of telemedicine and, at the same time, reinforced the need to define a common standard for this practice. This was initially confirmed in the State-Regions Agreement of 17 December 2020 with the approval of the “National Indications for the provision of services in Telemedicine” [5,6,7].

The objective of this article is to illustrate the regulatory evolution of the last decade in the field of telemedicine in Italy, as well as its regulatory aspects and its impact in the ethical sphere.

## 2. Evolution of Telemedicine in Italy

Italy is a European country with one of the highest rates of demographic aging and one of the fastest rates of growth in life expectancy, and experiences in the international arena show how the field of chronicity can achieve concrete and substantial benefits from the use of ICT technologies, including telemedicine. The goal of regulatory interventions in this area is to overcome the fragmentation present throughout the country. Telemedicine is often limited to projects of an experimental nature; thus, to define a model of shared governance of the various initiatives, unifying the guidelines and application models of these services and ensuring their interoperability are necessary actions to take in order for telemedicine to become a tool that is utilized throughout the country in a structured way.

There are, essentially, four most relevant policies which have been implemented in order to increase the diffusion and efficiency of telemedicine services in Italy: the first, National Guidelines for Telemedicine, acknowledged in the Agreement between the Government, the Regions, and Autonomous Provinces in 2014; the Digital Growth Strategy 2014–2020, a document aimed at pursuing the digitization objectives proposed by the European Commission; the National Directives for the Delivery of Telemedicine Services, a document published at the end of 2020 by the Permanent Conference for Relations between the State, the Regions, and the Autonomous Provinces to promote the digital evolution of medicine in light of the COVID-19 pandemic; the National Recovery and Resilience Plan (PNRR), part of the Next Generation EU (NGEU) program, a package worth EUR 750 billion which was agreed upon by the European Union in response to the pandemic crisis.

### 2.1. National Guidelines for Telemedicine (State-Regions Agreement 2014)

The first significant intervention at the central level in the field of telemedicine dates back to 2010. A working group composed of experts from the Ministry of Health and the Higher Health Council was established by the Minister of Health, who drafted the first National Guidelines for Telemedicine and transposed the Agreement between the Government, the Regions, and the Autonomous Provinces in 2014. To define telemedicine, the document highlights how it “does not replace traditional healthcare delivery in the doctor-patient relationship, but complements it to potentially improve effectiveness, efficiency and appropriateness. Telemedicine must therefore comply with all the rights and obligations inherent in any healthcare act” [8]. The document aims to identify the purposes, possible areas of application, and main categories of services that can be provided in telemedicine. It also identifies ways to charge for the services themselves, in addition to covering regulatory and ethical aspects.

Specifically, areas of application for telemedicine in both health and social-health contexts are identified (e.g., continuity of care and hospital-territory integration; relevant pathologies; emergency–urgency system; reorganization of laboratory and imaging diagnostics), as are the macro-categories that can be associated with telemedicine services, including specialist telemedicine (e.g., telehealth, teleconsultation/telecooperation to be used for the management of any pathology); telehealth related mainly to primary care and chronicity management; and telehealth in the social-health sphere, aimed at elderly, frail, or disabled persons.

Additionally, the need to implement a systematic approach based on a regional governance model of telemedicine initiatives is recalled through the establishment and maintenance of a Regional Catalog containing active, planned, and concluded telemedicine initiatives, projects, and services. Regional planning that takes into account the specific needs of the territory and identifies services/performances that can be improved and optimized through telemedicine solutions on that basis is also essential. This approach allows for the evaluation of regional initiatives at the national level, enabling the comparison of the results obtained by various regions.

Finally, this document identifies some aspects relevant to the widespread and structured use of telemedicine, including the need for training aimed at both the citizen/patient and the healthcare providers and technicians; the proper management of the patients’ personal data in compliance with the principles of confidentiality, integrity, and availability; and the provision of adequate information to patients, respecting ethical implications (safeguarding the dignity of the patient and ethical certification) with the acquisition of their consent. The clear identification of responsibilities, tasks, and functions to be assigned that are consistent with current regulations and appropriate organizational and technological solutions will make it possible to maintain the availability of information only to those who are entitled to its use.

### 2.2. Digital Growth Strategy 2014–2020 and Three-Year Plan for Information Technology in Public Administration

The Strategy for Digital Growth 2014–2020 identified several activities aimed at creating fully integrated solutions in telemedicine. These activities involve strong interactions between health, business, and hospital information systems, as well as the widespread use of cloud technologies. The Strategy also emphasizes the importance of applying criteria to standardize the collection and processing of health data. These interventions are considered fundamental for implanting innovative organizational models capable of delivering services to patients and operators while also supporting territorial social-health activities, such as facilitating diagnostics, supporting care pathways, and managing chronic conditions. Telemedicine, telemonitoring, and teleconsultation, which require the use of innovative electromedical instruments, sensors, video communication software, and other equipment both for remote control of the patient and to facilitate conversations between patients and healthcare providers, will finally be developed and widely disseminated [9].

The document also draws attention to an additional digital health tool, the Electronic Health Record. Specifically, the European Commission intends to proceed with the implementation of an Electronic Health Record (EHR) for citizens, understood as a set of clinical documents (patient summary, reports, prescriptions, etc.) pertaining to one’s health status and arising from one’s relationship with the various actors of the National Health Service. In addition, the outcome of a survey promoted in 2019 by the Ministry of Health and conducted by a working group as part of the NSIS Steering Committee appears to be interesting in this regard. A questionnaire designed to survey all active telemedicine initiatives in Italy was submitted to the various regions in 2018. As a result of this survey, 282 initiatives were discovered, the distribution of which highlighted significant fragmentation and interregional inhomogeneity. The main medical specialties were cardiology and radiology [10].

### 2.3. National Directives for the Delivery of Telemedicine Services (State-Regions Agreement 2020)

In the midst of the SARS-CoV-2 pandemic, with the need to ensure as much social distancing as possible and to reduce the pressure on healthcare facilities (particularly hospitals), the State-Regions Agreement approving the National Directives for the Delivery of Telemedicine Services was signed on 17 December 2020. The advent of the pandemic highlighted a less-than-positive balance sheet concerning the effectiveness of the 2014 National Guidelines for Telemedicine [8], which, until then, represented what was essentially the only regulatory core at the national level, and primarily consisted of soft law.

It should also be pointed out that, after the issuance of these guidelines, two important pieces of legislation were passed: law 219/2017 on informed consent and Advance Treatment Arrangements (DAT), relevant in the event of possible and future incapacity to self-determine; and EU Regulation 2016/679, the General Data Protection Regulation (hereafter “GDPR”), for the protection of individuals with regard to the processing of personal data and the free movement of such data. Therefore, the 2014 guidelines, in light of the new European privacy legislation, were insufficient to guarantee the security of personal data and clinical data processed through electronic tools [11]. In the State-Regions 2020 Agreement [12], the national directives mentioned above identify clear and uniform regulatory bases throughout the country for certain specific areas of telemedicine, such as telehealth, medical teleconsultations, teleconsultations by health professionals, and telereferrals.

Telemedicine services, with respect to appropriateness of delivery, are differentiated into four types:

- Services that can be assimilated into any traditional diagnostic and/or therapeutic healthcare service, representing an alternative delivery;
- Services that cannot replace traditional healthcare delivery, but support it by making it more accessible and/or increasing its efficiency and distribution equity;
- Services that supplement traditional delivery in varying proportions, making it more effective and more able to adapt dynamically to changes in patients’ care needs;
- Services that prove capable of completely replacing traditional healthcare delivery, representing new diagnostic and/or therapeutic methods and/or techniques and implementing new care practices that are useful to patients.

The document provides a set of rules related to the remuneration/tariff system, prescribing, booking, and reporting. Regarding the minimum elements of functionality required for a remote delivery service, the following are vital: compatibility with the GDPR in terms of the processing of personal data, for which an in-depth discussion is presented in the section below; certification of the telemedicine system as a medical device; and appropriate classification with respect to the diagnostic/therapeutic purposes offered through the remote service [13].

The minimum service standards for the provision of telemedicine services are also defined, including:

- The designation of managers in the technical and health-related organization of telemedicine pathways, and in the management and maintenance of the platforms used to implement these pathways;
- The provision of clear and comprehensive information to citizens, within the Service Charter, on the types of services that can be provided through telemedicine and their timing, as well as costs and access methods;
- The guarantee of user access to data acquired through the regional infrastructures of the Electronic Health Record and Online Referral Collection;
- The presence of an adequate training plan for telemedicine service delivery centers and users (patients/caregivers).

It seems appropriate to highlight, finally, what has been specified with reference to healthcare liability, namely that “for the purposes of clinical risk management and health care liability, the correct attitude consists in choosing the operational solutions that from the medico-medical point of view offer the best guarantees of proportionality, appropriateness, effectiveness and safety while respecting the rights of the person. […] all the legislative and deontological norms proper to the health professions, as well as the guiding documents of bioethics, apply to health activities in telemedicine. Finally, it remains in the responsibility of the healthcare provider to evaluate, at the end of a service provided at a distance on the degree of achievement of the objectives that the service itself was intended and that is, in case of insufficiency of the result for any reason (technical, related to the conditions encountered by the patient or other) the obligation of rescheduling the service in the presence” [14].

### 2.4. National Recovery and Resilience Plan-Mission 6-Health

If the desire for clear and standardized regulation at the national level is expressed by the National Indications, the National Recovery and Resilience Plan (PNRR) represents a tangible commitment by the government to promote and enhance digital health, including telemedicine. As a plan consisting of investments and reforms for the use of funds allocated by the Next Generation EU, the PNRR was approved in July 2021 by the Council of the European Union. The PNRR has set ambitious goals under Mission 6-Health, which will receive EUR 15.63 billion in funding, including EUR 7 billion earmarked for strand M6C1-Proximity networks, facilities, and telemedicine for territorial healthcare.

The COVID-19 pandemic highlighted some important criticalities of the National Health Service (NHS). This is a consequence of two main factors: first, a decade of public spending containment policies, with horizontal cuts in the resources made available to the NHS rather than interventions aimed at eliminating or reducing waste and reorganizing the distribution of resources; and second, to the process of corporatization initiated by the Legislative Decree 502/92 [15] that has progressively oriented the choices of NHS entities toward efficiency and productivity objectives, leading to a progressive impoverishment of some sectors, the first and foremost of which are hospital-territory integration and social healthcare. There are already significant territorial gaps known to the public, further amplified by the pandemic, which also need to be addressed and overcome in the immediate future in order to ensure continuous and diversified care based on health status to all patients throughout the country in a uniform and homogeneous manner.

The M6C1 mission aims precisely to strengthen the standard of care in the territory. In light of the demographic, epidemiological, and social trends taking place in our country, telemedicine represents within this mission one of the main tools that is intended to be developed to strengthen home-based services (the “Home as the first place of care”). This will be achieved through the identification of a shared model for the delivery of home care that makes the most of the possibilities offered by new technologies (telemedicine, home automation, digitalization, etc.) and through the use of telemedicine to support chronic conditions. Overall, the resources allocated by the PNRR for telemedicine amount to EUR 1 billion, and these funds will be used to finance telemedicine projects proposed by the regions based on the priorities and guidelines of the Ministry of Health. It seems appropriate to highlight what is specified in this regard within the PNRR, namely that “to obtain funding, the projects will first of all have to be able to integrate with the Electronic Health Record, achieve quantitative performance targets related to the main objectives of telemedicine and the National Health System, as well as ensure that their development results in an effective harmonization of health services. Indeed, preference will be given to projects that insist on multiple regions, leverage existing successful experiences, and aspire to build true “telemedicine platforms” that are easily scalable” [16].

As part of the work aiming to implement these policies, we note the recent publication in the Official Gazette of the Decree of the Minister of Health, 29 April 2022, approving the organizational guidelines: the “Digital Model for the implementation of home care, for the purpose of achieving EU Milestone M6C1-4 of the Plan for Italy’s Recovery and Resilience” [17]. The guidelines define a reference organizational model for the implementation of various telemedicine services in the home setting. Through the identification of innovative processes for taking care of patients at home, as well as the enhancement of multiprofessional and multidisciplinary collaboration, the Regions and Autonomous Provinces will have to adapt and take into account territorial specificities.

With regard to the Electronic Health Record (EHR), recent years have seen an important evolution. the Three-Year Plan for Information Technology in Public Administration 2020–2022 (the subject of a recent update) includes, among its main objectives in the health sector, increasingly feeding and digitizing the EHR with health documents provided by territorial health facilities. This will be accomplished through lines of action such as updating the interoperability specifications between regional electronic health records (an activity concluded with publication in February 2022 of the new specifications) and adapting the national EHR-INI (Electronic Health Record—National Interoperability Infrastructure) platform to these new interoperability specifications.

The presence of a structured EHR is also a strength for the development and interoperability of telemedicine tools, as the integration of telemedicine platforms with the Electronic Health Record would ensure the full and complete accessibility of all health data and information acquired through telemedicine tools (e.g., telemonitoring, televisit, etc.) at any time by both the citizen and the various health professionals involved in the care process in various capacities.

### 2.5. Ethical and Regulatory Aspects Related to the Provision of Telemedicine Services

As highlighted in the previous paragraphs, the regulatory interventions promoted at the central level, first with the National Guidelines of 2014, then more markedly in the 2020 National Directives, have focused attention on the need to ensure regulatory compliance with regard to cybersecurity and processing of personal data in the development and implementation of telemedicine tools. In this sense, the EU Regulation 2016/679 [18], which entered into force definitively on 25 May 2018, is the main regulatory reference. It has introduced, compared to the previous discipline, a new approach to the protection and processing of personal data based on the principles of accountability and risk management. Indeed, Article 32 stipulates that the controller must take appropriate measures to demonstrate compliance by identifying the risk associated with the processing; assessing it in terms of its origin, nature, likelihood, and severity; and identifying the best practices to mitigate the risk.

A GDPR-compliant telemedicine platform must, therefore, provide:

- The ability to manage security and data protection as early as the design phase of the application (so-called privacy by design);
- A clear and complete identification of the figures responsible for data processing within the structure providing the telemedicine service;
- A method of acquiring informed consent from the patient (or relative/guardian in the case of a minor patient) and accessing the patient’s prior information (Articles 13 and 14) containing indications on the purposes of the processing; the categories of personal data processed; the recipients/categories of recipients of the acquired data; the period of data retention; and the indication of all the rights of the data in relation to consent/denial of processing, changes to consent, access to personal data, portability, and the right to be forgotten;
- The adoption of organizational and technical security measures aimed at ensuring that the acquired data are processed and stored in accordance with the principles of integrity, confidentiality, availability, minimization, accuracy, updating, and limitation in a form that allows the identification of data subjects for a period of time not exceeding the achievement of the purposes for which they are processed. This includes, for example, all measures to ensure the confidentiality of data (e.g., encryption, pseudonymization), protection from cyber-attacks, as well as all business continuity and disaster recovery systems aimed at ensuring maximum data availability and uptime of the platforms.

EU Regulation 2016/679 is characterized by a strong ethical perspective. In fact, Article 4 states that “the processing of personal data should be at the service of man”. This regulation, while recognizing the enormous contribution of technology to economic and social progress, highlights the need for it to be developed in a responsible manner and for individuals to always have control over their personal data, by virtue of the principle that a person’s dignity is a condition proper to the human being that precedes and underlies every right. A further ethical implication of the introduction of telemedicine tools is the delocalization of healthcare delivery, by which subjects are freed from the need for physical co-presence, and the traditional approach to the doctor–patient relationship is thus substantially altered.

The evolution (or revolution) of technologies associated with ICT often travels at speeds considerably faster than human adaptability [19]. Therefore, it seems essential that the spread of telemedicine practices be accompanied by a careful assessment of the inevitable impact on the bioethical sphere and related issues. As recalled in the 2014 National Guidelines, the risk of alteration in the perception of the quality of care by the person in need of that care highlights, first and foremost, the need for the provider to invest in information before the patient does so, “even devoting the necessary time to meet information needs well beyond informed consent, which today is sometimes interpreted in a defensive logic and not one of dialogue and sharing with the patient” [8].

Technology has now become an integral and indispensable part of the process of care. Increasingly often, and in an increasingly cumbersome way, it stands between doctor and patient; therefore, careful governance and control of technology appear to essential so that it does not lead to fractures or excessive vulnerability in the doctor–patient relationship and does not cause one to lose sight of the human aspects of healthcare provision. Listening to and observing the patient constitute important facets of care, and also provide useful information for elaborating on the diagnosis and identifying the most appropriate therapeutic path. Therefore, referring to the increasing diffusion of information technology, we agree with the following statement by Fasan: “if today technology still allows us to float while facing the perfect storm, it must not be what will sink us in the near future” [20].

## 3. Conclusions

Numerous challenges have been faced in the attempt to regulate telemedicine in Italy from a regulatory point of view, just as many efforts have been made by governmental administrations to build a solid and coherent legal framework. Defining a sufficiently comprehensive regulatory scenario is made complex both by the very rapid evolution of technology and the constant evolution of European cybersecurity regulations. These difficulties are compounded by critical issues related to cultural and organizational factors, such as doctors’ and patients’ lack of awareness of the technology behind telemedicine and resistance to technological change.

Therefore, it seems possible to conclude that numerous challenges still lie on the horizon. This is in spite of the fact that over the last ten years, significant progress has been made in laying the regulatory foundations for an increasingly effective telemedicine program that is both integrated into the dynamics of the healthcare system at the regional and local level and respectful of patients’ rights in terms of diagnosis and treatment.

The very likely further evolution and widespread diffusion of telemedicine services that will be witnessed in the coming years will, in fact, have to come to terms with regulatory evolution. This system will have to be able to reconcile technological progress with the standardization of services throughout the country, the guarantee of data security, the provision of adequate training programs for healthcare users, and monitoring programs for telemedicine services.
